# Infantile-onset inflammatory bowel disease in a patient with Hermansky-Pudlak syndrome: a case report

**DOI:** 10.1186/s12876-019-0929-9

**Published:** 2019-01-11

**Authors:** Jun Ishihara, Tatsuki Mizuochi, Takashi Uchida, Yugo Takaki, Ken-ichiro Konishi, Masahiko Joo, Yasuhiko Takahashi, Shinichiro Yoshioka, Hironori Kusano, Yoji Sasahara, Yushiro Yamashita

**Affiliations:** 1 Department of Pediatrics and Child Health, Kurume University School of Medicine, 67 Asahi-machi, Kurume, 830-0011 Japan; 2 Department of Pediatrics, Tohoku University Graduate School of Medicine, Sendai, Japan; 3 Department of Pediatrics, Japan Community Healthcare Organization, Kyushu Hospital, Kitakyushu, Japan; 4 Division of Gastroenterology, Department of Medicine, Kurume University School of Medicine, Kurume, Japan; 5 Department of Pathology, Kurume University School of Medicine, Kurume, Japan

**Keywords:** Hermansky-Pudlak syndrome, Early-onset inflammatory bowel disease, Monogenic inflammatory bowel disease, Infliximab

## Abstract

**Background:**

Hermansky-Pudlak syndrome (HPS) is a rare, genetically heterogeneous disorder that manifests oculocutaneous albinism together with bleeding diatheses that reflect a platelet storage pool deficiency. Ten genetic subtypes of this autosomal recessive condition have been described to date. Some patients with Hermansky-Pudlak syndrome type 1, 4, or 6 develop Crohn’s-like inflammatory bowel disease at any age including early childhood, but most often in adolescence or young adulthood. Here we report infantile-onset of inflammatory bowel disease in a patient with Hermansky-Pudlak syndrome type 1 who responded to infliximab.

**Case presentation:**

A Japanese boy, the second child of non-consanguineous healthy parents, was born with chalky white skin, silvery-white hair, and gray eyes, representing oculocutaneous albinism. He developed frequent diarrhea and fever accompanied by weight loss at 6 months, and was diagnosed with Crohn’s-like inflammatory bowel disease based on the endoscopic finding of longitudinal ulcerations in the colon and the histopathologic finding of nonspecific chronic inflammation without granulomas at the age of 11 months. Treatment with an elemental diet, salazosulfapyridine, and corticosteroids failed to improve clinical or laboratory abnormalities, and the diarrhea became bloody. At 13 months he began treatment with infliximab, which produced marked improvement followed by clinical remission. Endoscopy at 20 months demonstrated healing of the colonic mucosa. At 22 months he is in sustained clinical remission receiving only infliximab. Because albinism with inflammatory bowel disease suggested Hermansky-Pudlak syndrome, we performed genetic screening using next-generation sequencing in a targeted gene panel analysis for primary immunodeficiency disease and/or inflammatory bowel disease. The patient proved to have a compound heterozygous mutation of the *HPS1* gene resulting in Hermansky-Pudlak syndrome type 1.

**Conclusions:**

We consider this report to be the first account of type 1 Hermansky-Pudlak syndrome with infantile-onset of inflammatory bowel disease. Early administration of infliximab was effective. We recommend next-generation sequencing for patients with very early-onset inflammatory bowel disease suspected to be monogenic.

**Electronic supplementary material:**

The online version of this article (10.1186/s12876-019-0929-9) contains supplementary material, which is available to authorized users.

## Background

Hermansky-Pudlak syndrome (HPS) is a rare, genetically heterogeneous disorder that manifests oculocutaneous albinism together with bleeding diatheses that reflect a platelet storage pool deficiency. Electron microscopically, lysosomes and related organelles in reticuloendothelial and other organs have shown lipofuscin accumulation [1, 2]. Ten genetic subtypes of this autosomal recessive condition have been described to date [1, 3]. Some patients with HPS, specifically those with genotype HPS-1, HPS-2, or HPS-4, are predisposed to interstitial lung disease that usually presents in mid-adulthood but rarely in late adolescence [1]. In addition, some patients with HPS, specifically HPS-1, − 4, and − 6, develop Crohn’s-like inflammatory bowel disease (IBD). HPS with IBD can present at any age including early childhood, but most often emerges in adolescence and young adulthood [1, 2].

We report an infantile-onset IBD patient with HPS-1 who responded to infliximab (IFX).

## Case presentation

A Japanese boy was born by spontaneous vaginal delivery at 39 weeks of gestation, with a birth weight of 2616 g. He was the second child of non-consanguineous healthy parents.

He was born with chalky white skin, silvery-white hair, and gray eyes, indicating oculocutaneous albinism. He also showed horizontal nystagmus. At 6 months of age, he developed frequent diarrhea and fever accompanied by weight loss.

**Fig. 1:**
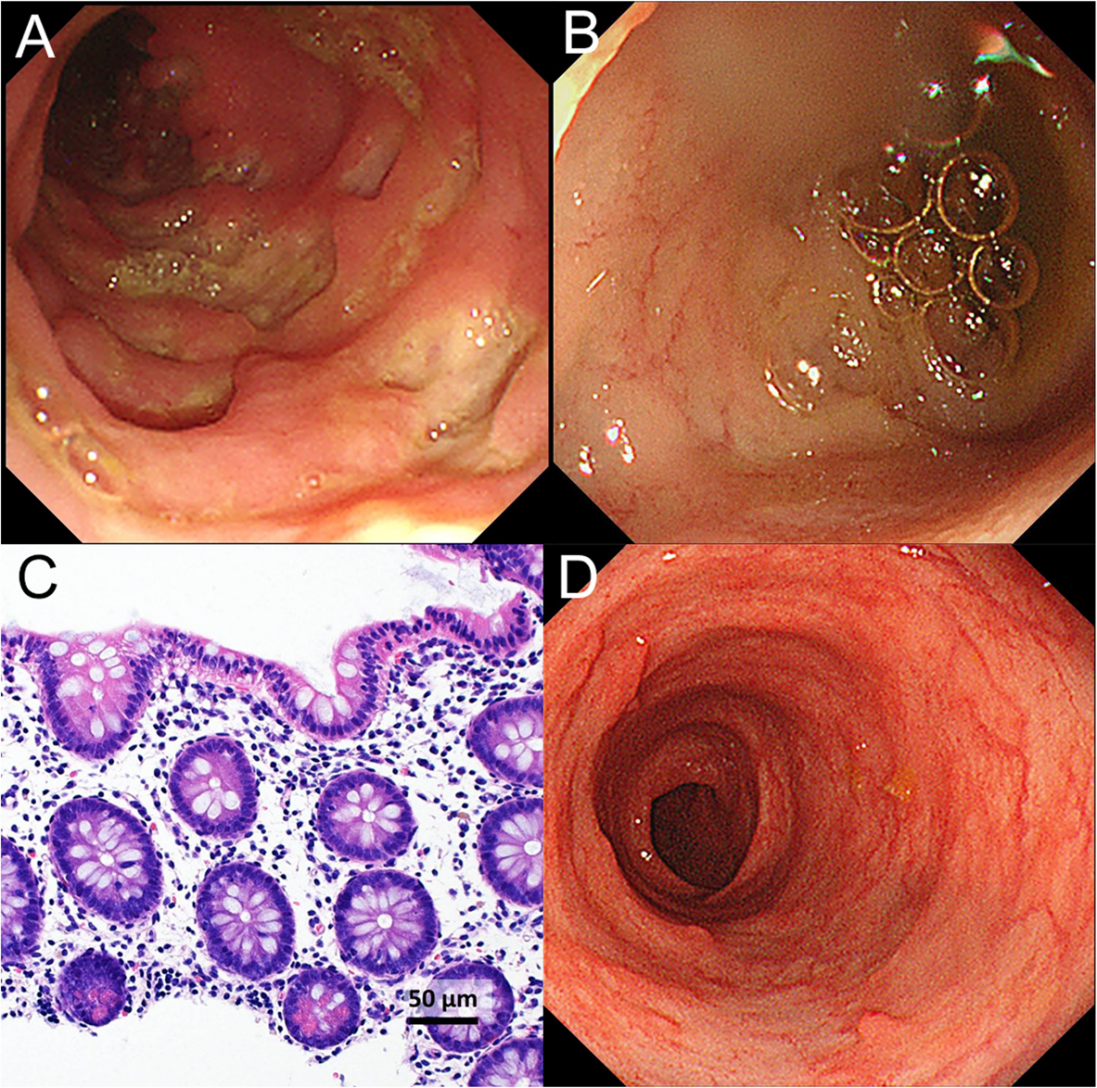
Endoscopic and histopathologic findings before and after infliximab. Endoscopy before infliximab treatment showed erosive changes, multiple aphthous ulcers, and longitudinal ulcerations in the colon (**a**) but normal mucosa in the terminal ileum (**b**). Endoscopic biopsy specimens from the colon showed mild lymphoplasmacytic infiltration in the lamina propria, and Paneth cell metaplasia (**c**; hematoxylin and eosin stain). Endoscopy 7 months after initiation of infliximab treatment showed mucosal healing in the colon (**d**)

At 11 months of age, he was hospitalized at Kyushu Hospital. Laboratory results included a white blood cell count of 20.3 × 10^9^/L (normal range, 4.4–19.1); red blood cell count, 534 × 10^10^/L (380–523); hemoglobin, 92 g/L (100–142); platelet count, 83.6 × 10^10^/L (22.0–76.0); serum total protein, 66 g/L (53–72); and albumin, 31 g/L (32–48). C-reactive protein was 104.5 mg/L (< 1.4) and erythrocyte sedimentation rate, 71 mm/hr. (< 10). Prothrombin time was 14.6 s (11.0–15.0); activated partial thromboplastin time, 31.4 s (24.0–39.0); and bleeding time, 2.3 min (2.0–5.0). Abdominal ultrasonography and computed tomography showed bowel wall thickening in the transverse and descending colon. Endoscopy showed erosive changes, multiple aphthous ulcers, and longitudinal ulcerations in the colon, but normal mucosa in the terminal ileum (Fig. 1a and b); endoscopic biopsy specimens from the colon showed nonspecific chronic inflammation without granulomas (Fig. 1c).

Based on clinical, laboratory, and endoscopic findings, the patient was diagnosed with Crohn’s-like IBD. He was treated with an elemental diet, salazosulfapyridine, and corticosteroids, which failed to improve clinical or laboratory findings, and bloody diarrhea ensued. Given lack of improvement, he was transferred at 13 months of age to Kurume University Hospital, where he began treatment with IFX. He soon showed marked improvement, followed by clinical remission. At 20 months of age, endoscopy showed healing of the colonic mucosa (Fig. 1d). Currently, at 22 months of age, the patient is in sustained clinical remission, receiving only IFX (7 mg/kg) every 8 weeks.

Because of albinism, nystagmus, and IBD, an association suggestive of HPS, platelet morphology was evaluated. Electron microscopy showed absence of dense bodies in platelets, which was consistent with a diagnosis of HPS. Following parental informed consent, we performed genetic screening using next-generation sequencing in a targeted gene panel analysis for primary immunodeficiency disease and/or IBD including *HPS1*, *HPS4*, and *HPS6* genes (Additional file 1) [1, 4]. We identified 2 heterozygous mutations in the *HPS1* gene in the patient (c.398 + 5G > A and c.1323dupA) and a heterozygous mutation in each parent (father, c.398 + 5G > A; mother, 1323dupA). These mutations have been reported previously in Japanese patients with HPS-1 [5]. We diagnosed the patient with HPS-1 resulting from compound heterozygous mutation of the *HPS1* gene.

## Discussion and conclusions

HPS can be caused by 10 different genotypes involving various chromosomes. These aberrant genes cause abnormal vesicle formation involving melanosomes, platelet dense bodies, and a subset of lysosomes. This results in visual impairment, skin hypopigmentation, and increased risk of bleeding [1, 3]. IBD has been reported in patients with HPS-1, − 4, and − 6; the pathogenetic mechanisms are unknown [1, 2].

IBD in HPS shows many of the same pathologic features as the more common Crohn’s colitis: irregular distribution of large bowel lesions, interspersed with regions of normal mucosal architecture; superficial crypt abscesses; and prominent inflammatory cell infiltrates in involved areas [2, 6, 7]. In a study of 122 subjects with HPS, 8% overall were found to have IBD; among those with gastrointestinal symptoms, 33% were diagnosed with IBD [2]. IBD in HPS can present at any age including early childhood but most commonly becomes evident in adolescence and young adulthood. To our knowledge, the youngest HPS patient reported to develop IBD was 2 years old [2], making our patient the youngest to present with IBD and the first reported HPS patient with IBD onset during infancy.

In some patients with HPS, conventional therapy of IBD including aminosalicylates and corticosteroids has been unsuccessful. Recent reports maintain that immunosuppressants and anti-tumor necrosis factor α therapy such as IFX may be effective for IBD in patients with HPS [2, 6, 7]. Use of compounds containing 5-aminosalicylic acid is an area of controversy because of the HPS platelet storage pool defect [1]. In fact, our patient is now in sustained clinical remission with colonic mucosal healing using only IFX.

Patients with a diverse spectrum of rare genetic disorders can present with IBD, a situation referred to as monogenic IBD [4, 8, 9]. Patients with these disorders often develop symptoms during infancy or early childhood, along with endoscopic or histologic features of Crohn’s disease, ulcerative colitis, or unclassified IBD. HPS is considered one of the monogenic IBDs [1, 2, 8]. Distinguishing monogenic forms among IBD patients under 6 years old can be crucial in determining the best treatment. Genetic screening using next-generation sequencing is a highly useful approach to diagnosis of patients with monogenic IBD [4, 8, 9]. In this patient, the clinical features of oculocutaneous albinism with nystagmus are consistent with an underlying diagnosis of HPS [1]. Such characteristic features may aid in diagnosing specific etiologies of infantile-onset IBD. Our patient represents a case in point; we diagnosed him with HPS-1 by genetic analysis as an infant and could treat him successfully with early introduction of IFX.

In conclusion, we consider our patient to be the first known HPS-1 patient with infantile-onset of IBD. Early introduction of IFX was effective therapy. Physicians should consider genetic screening using next-generation sequencing when they treat patients with very early-onset IBD suspected to be monogenic.

## Additional file

Additional file 1: List of genes responsible for pediatric IBD analyzed in this study (adapted from Reference 4). (DOCX 29 kb)

## Abbreviations

HPS: Hermansky-Pudlak syndrome
IBD: Inflammatory bowel disease
IFX: Infliximab
