# Supplementary material for: Infantile-onset inflammatory bowel disease in a patient with Hermansky-Pudlak syndrome: a case report
Source: BMC Gastroenterol. 2019 Jan 11;19:9. doi: 10.1186/s12876-019-0929-9 (PMC6329123; doi:10.1186/s12876-019-0929-9)
Supplement: Supplementary file 1 — List of genes responsible for pediatric IBD analyzed in this study (adapted from Reference 4). (DOCX 29 kb) [file 12876_2019_929_MOESM1_ESM.docx]

**Additional file 1: List of genes responsible for pediatric IBD analyzed in this study (adapted from Reference 4).**

**Disease classification Syndrome/Disorder Gene Inheritance**

Immunodeficiencies γc deficiency IL2RG XL

affecting cellular and RAG1 deficiency RAG1 AR

humoral immunity RAG2 deficiency RAG2 AR

Artemis deficiency DCLRE1C 　AR

ADA deficiency ADA AR

DNA ligase Ⅳ deficiency LIG4 AR

CD3γ deficiency CD3G AR

ZAP70 deficiency ZAP70 AR

DOCK8 deficiency DOCK8 AR

LRBA deficiency LRBA AR

IL-21 deficiency IL21 AR

CD40 ligand deficiency CD40LG XL

Combined Wiskott-Aldrich syndrome WAS XL

immunodeficiency with Dyskeratosis congenita DKC1 XL

associated or syndromic Hoyeraal-Hreidarsson syndrome RTEL1 AR

features　　 XL-EDA-ID IKBKG XL

EDA-ID IKBA AD

Immunodeficiency with multiple intestinal atresia TCC7A AR

Predominantly antibody X-linked agammaglobulinemia BTK XL

deficiencies AID deficiency AICDA AR

Common variable immunodeficiency ICOS AR

Phosphatidylinositol 3-kinase deficiency PIK3R1 AR

Diseases of X-linked lymphoproliferative syndrome type 1 SH2D1A XL

immune dysregulation X-linked lymphoproliferative syndrome type 2 XIAP XL

Immune dysregulation, polyendocrinopathy, FOXP3 XL

enteropathy, X-linked syndrome

IL-10 deficiency IL10 AR

IL-10Rα deficiency IL10RA AR

IL-10Rβ deficiency IL10RB AR

NFAT5 haploinsufficiency NFAT5 AD

Familial hemophagocytic lymphohistiocytosis type 5 STXBP2 AR

CD25 deficiency IL2RA AR

Hermansky-Pudlak syndrome HPS1 AR

Hermansky-Pudlak syndrome HPS4 AR

Hermansky-Pudlak syndrome HPS6 AR

Congenital defects of Chronic granulomatous disease CYBB 　 XL

phagocyte number, Chronic granulomatous disease CYBA AR

function, or both Chronic granulomatous disease NCF1 AR

Chronic granulomatous disease NCF2 AR

Chronic granulomatous disease NCF4 AR

Glycogen storage disease type 1b G6PT1 AR

Leukocyte adhesion deficiency type 1 INTGB2 AR

Severe congenital neutropenia type 4 G6PC3 AR

Defects in intrinsec STAT1 deficiency STAT1 AD

and innate immunity Phospholipase C-γ2 deficiency PLCG2 AD

Auto-inflammatory Familial Mediterranean fever MEFV AR

disorders MVK deficiency MVK AR

Inflammatory skin and bowel disease-1 ADAM17 AR

Complement deficiencies MASP2 deficiency MASP2 AR

Others Trichohepatoenteric syndrome SKIV2L AR

Trichohepatoenteric syndrome TTC37 AR

Dystrophic bullosa COL7A1 AR

Kindler syndrome FERMT1 AR

Familial diarrhea GUCY2C AD

Tufting enteropathy EPCAM AR

Hirschsprung’s disease RET AD

AD, autosomal dominant; AR, autosomal recessive; XL, X-linked; RAG, recombination activating gene; ADA, adenosine deaminase; CD, cluster of differentiation; ZAP, zeta-chain associated protein kinase; DOCK, dedicator of cytokinesis; LRBA, lipopolysaccharide-responsive and beige-like anchor protein; EDA-ID, anhidrotic ectodermal dysplasia with immunodeficiency; AID, activation-induced cytidine deaminase; NFAT, nuclear factor of activated T cells; STAT, signal transducers and activator of transcription; MVK, mevalonate kinase; MASP, mannose-binding lectin-associated serine protease.
